# Supplementary material for: Saccharomyces boulardii (CNCM I-745) improves intestinal damage in sepsis by remodeling intestinal flora structure
Source: Front Cell Infect Microbiol. 2025 Jul 31;15:1602792. doi: 10.3389/fcimb.2025.1602792 (PMC12350329; doi:10.3389/fcimb.2025.1602792)
Supplement: Supplementary file 1 [file DataSheet1.zip › summary/summary/7_Differential_analysis/1_diff_abundance/7_Species/Group.AW4_vs_AW0/Group.AW4_vs_AW0_diff_barplot.pdf]

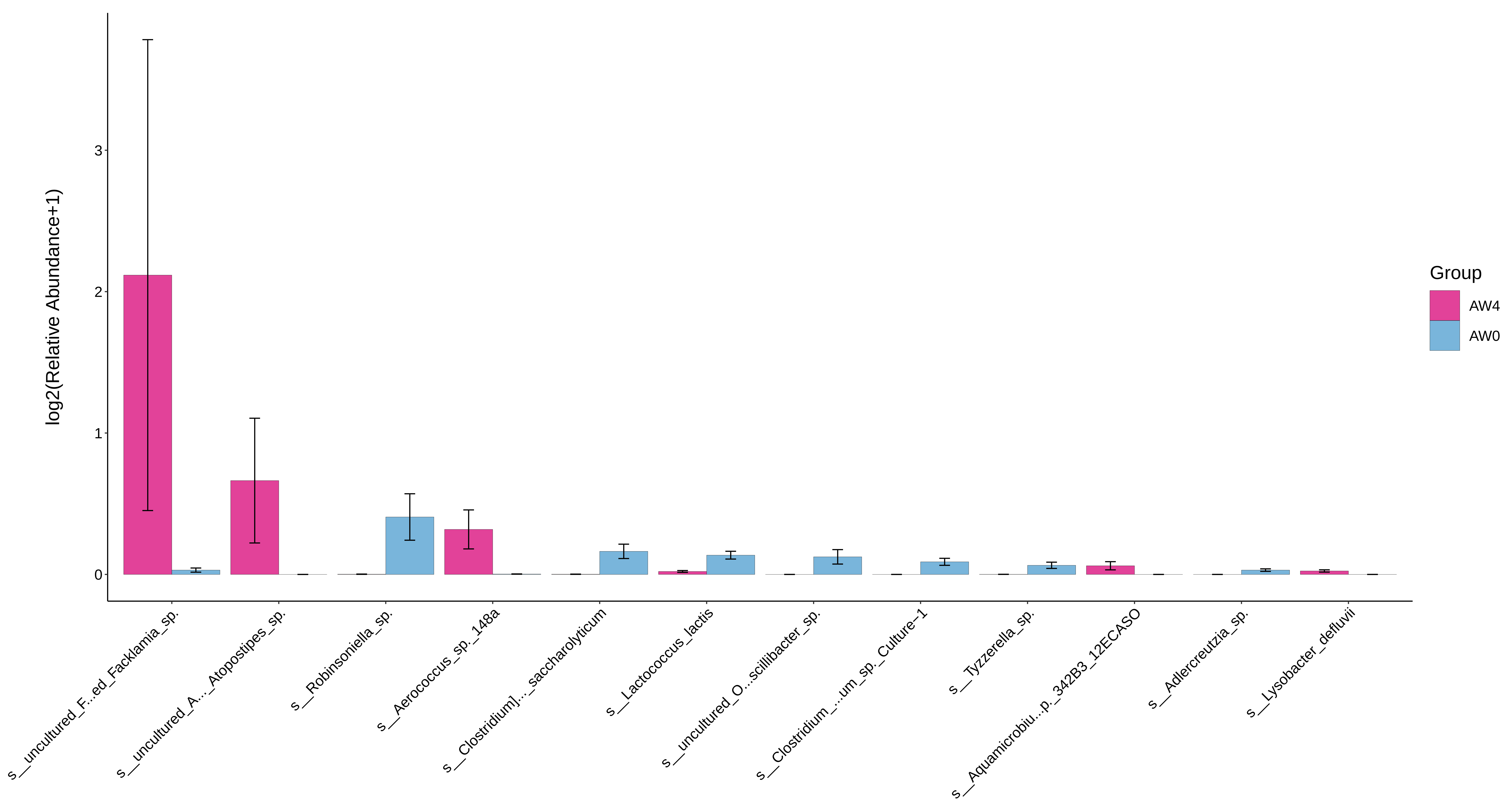

log2(Relative Abundance+1)
0
1
2
3
Group
AW4
AW0
s__uncultured_F...ed_Facklamia_sp.
s__uncultured_A..._Atopostipes_sp.
s__Robinsoniella_sp.
s__Aerococcus_sp._148a
s__Clostridium]..._saccharolyticum
s__Lactococcus_lactis
s__uncultured_O...scillibacter_sp.
s__Clostridium_...um_sp._Culture−1
s__Tyzzerella_sp.
s__Aquamicrobiu...p._342B3_12ECASO
s__Adlercreutzia_sp.
s__Lysobacter_defluvii
